# Substantially Improved Electrofusion Efficiency of Hybridoma Cells: Based on the Combination of Nanosecond and Microsecond Pulses

**DOI:** 10.3390/bioengineering9090450

**Published:** 2022-09-07

**Authors:** Meng Wu, Qiang Ke, Jinhao Bi, Xinhao Li, Shuheng Huang, Zuohua Liu, Liangpeng Ge

**Affiliations:** 1 College of Veterinary Medicine, Hunan Agricultural University, Changsha 410128, China; 2 Chongqing Academy of Animal Sciences, Chongqing 402460, China; 3 Nanjing Research Institute of Electronics Technology, Nanjing 210039, China; 4 State Key Laboratory of Power Transmission Equipment & System Security and New Technology, School of Electrical Engineering, Chongqing University, Chongqing 400044, China; 5 School of Nuclear Engineering, Purdue University, West Lafayette, IN 47906, USA; 6 College of Veterinary Medicine, Jilin Agricultural University, Changchun 130118, China; 7 School of Life Sciences, Westlake University, Hangzhou 310024, China; 8 College of Bioengineering, Chongqing University, Chongqing 400044, China; 9 Department of Biochemistry, University of Toronto, Toronto, ON M5S 1A8, Canada

**Keywords:** cell electrofusion, nanosecond/microsecond pulsed electric fields, equal energy, hybridoma cell

## Abstract

As the initial antibody technology, the preparation of hybridoma cells has been widely used in discovering antibody drugs and is still in use. Various antibody drugs obtained through this technology have been approved for treating human diseases. However, the key to producing hybridoma cells is efficient cell fusion. High-voltage microsecond pulsed electric fields (μsHVPEFs) are currently one of the most common methods used for cell electrofusion. Nevertheless, the membrane potential induced by the external microsecond pulse is proportional to the diameter of the cell, making it difficult to fuse cells of different sizes. Although nanosecond pulsed electric fields (nsPEFs) can achieve the fusion of cells of different sizes, due to the limitation of pore size, deoxyribonucleic acid (DNA) cannot efficiently pass through the cell pores produced by nsPEFs. This directly causes the significant loss of the target gene and reduces the proportion of positive cells after fusion. To achieve an electric field environment independent of cell size and enable efficient cell fusion, we propose a combination of nanosecond pulsed electric fields and low-voltage microsecond pulsed electric fields (ns/μsLVPEFs) to balance the advantages and disadvantages of the two techniques. The results of fluorescence experiments and hybridoma culture experiments showed that after lymphocytes and myeloma cells were stimulated by a pulse (ns/μsLVPEF, μsHVPEF, and control), compared with μsHVPEF, applying ns/μsLVPEF at the same energy could increase the cell fusion efficiency by 1.5–3.0 times. Thus far, we have combined nanosecond and microsecond pulses and provided a practical solution that can significantly increase cell fusion efficiency. This efficient cell fusion method may contribute to the further development of hybridoma technology in electrofusion.

## 1. Introduction

Cell fusion refers to using natural or artificial methods to make two cell types fuse into one cell, which displays the combined characteristics of both cells. Cell fusion plays an essential role in modern biotechnology. For example, one critical procedure in genetic engineering is introducing exogenous genetic material into a host cell. Such insertion of genes can be accomplished by fusing the host cell with a cell containing the desired genetic material. Furthermore, cell fusion plays a vital role in the production of monoclonal antibodies, which requires the fusion of antibody-producing cells with continuously dividing cancer cells such as myeloma cells [1,2,3,4,5].

Hybridoma technology was the earliest method to isolate monoclonal antibodies (mAbs) and is still used today [6,7,8]. In contrast to other mAb discovery techniques, once hybridoma cell clones are established, mAbs can be continuously secreted into culture supernatants or ascites collected from immunized mice for purification. Light and heavy chain combinations are not needed. The advent of this technology has accelerated the process of mAb research. Hybridoma technology relies primarily on mature B cells stored in secondary lymphoid organs. The process by which B cells undergo antigenic stimulation in peripheral lymphoid germinal centers is called somatic hypermutation (SHM) [9]. V(D)J rearrangement mechanisms of germline genes and SHM constitute the diversity of antibody complementarity-determining regions (CDRs) [10]. Hybridoma technology fuses these antigen-specific B cells with cells such as SP2/0 that can proliferate indefinitely, allowing B cells to be easily cultured in vitro and continue to secrete antibodies. In addition to common murine mAbs, hybridoma technology has also been applied to develop fully human antibody transgenic animals or direct human-derived antibodies [11,12,13].

The key to preparing hybridoma cells is efficient cell fusion. There are numerous methods to fuse cells, including chemical (polyethylene glycol, PEG) [14], viral (Hemagglutinating virus of Japan, HVJ) [15], and physical methods (electric fields) [16,17,18,19,20,21,22]. The latter one, electrofusion, is the most commonly used method owing to the highest repeatability and stability. There are three preconditions for cell electrofusion:

1. Cells should be arranged in close contact [20];
2. Electroporation should be created in the contact zone of cell membranes [21];
3. Cells should be alive—μsPEF was used in cell fusion technology by Zimmermann et al. as early as 40 years ago [22,23].

Nevertheless, in hybridoma technology, cell fusion based on μsPEF has considerable defects [24,25]. The production of antibodies primarily relies on the cell fusion of myeloma cells with B lymphocyte cells [21]. There are significant differences in the cell sizes (the radii of B lymphocytes and myeloma cells are 3.85 μm and 7.75 μm, respectively) [26]. According to the well-known Schwan equation [27], the transmembrane voltage (*TMV*) is defined as:

(1) $$TMV=1.5Ercos\theta \left(1-e^{\frac{-t}{\tau }}\right)$$

where *r*, *E*, *τ*, and *θ* are the cell radius, electric field, charging time constant of the cell membrane, and the angle between the electric field and the specified point, respectively. When the transmembrane potential reaches around 1 V, it is currently regarded as the potential threshold required to achieve electroporation [28]. The *TMV* of cell membranes induced by external microsecond pulses is proportional to the diameter of the cells. Accordingly, different-sized cells’ electrofusion by μsPEF faces a series of bottlenecks, the most significant of which is the low efficiency. When one tries to fuse cells of two different sizes, it is difficult to choose a proper field strength of the pulse for both cell types. When the field is only large enough to cause membrane electroporation in the larger cells (myeloma cells), it is not sufficient to induce a critical membrane potential to electroporate the membranes of the smaller cells (lymphocytes). On the other hand, if the field strength is increased to cause membrane electroporation in the smaller cell, the potential induced in the larger cell is so high that it will cause irreversible damage and destroy the cells [29]. Therefore, it is difficult to guarantee that two types of cells are in a suitable electroporation condition simultaneously, which will lead to low cell fusion efficiency when applying μsPEF, only 1% or less [21].

nsPEF has attracted increased attention in the biomedical field [30,31,32,33]. According to simulation and experimental research [34], different-sized cell fusion can be realized by nsPEF. The electroporation is mainly concentrated in the cell contact zone, and cell sizes have little impact on TMV when applying nsPEF [34]. According to the theory of cell electrofusion [35], not only the sufficient size but also a sufficient life span of pores is required to fuse cells. Although cell membranes can be electroporated by nsPEF, the radius of pores is only several nanometers, and they quickly disappear [36,37]. Therefore, when nsPEF is applied to electrofusion, some new problems arise (insufficient size and short life span of pores).

In order to achieve high cell electrofusion efficiency, a new pulse form needs to be put forward to overcome the bottlenecks caused by μsPEF or nsPEF mentioned above. Fortunately, in the study of gene transfer efficiency, S Guo et al. found that when compared with nsPEF, the higher gene transfer efficiency can be achieved by applying nsPEFs to cells first, followed by one low-voltage millisecond pulse electric field, but not in the opposite order [38]. Both gene transfection and cell electrofusion belong to reversible electroporation. Based on the research above, we propose a hypothesis—cell fusion efficiency can be improved by combining a nanosecond pulsed electric field with a low-voltage microsecond pulsed electric field. The purpose of this paper is to utilize the advantages of nsPEF and μsPEF. Firstly, nsPEF was used to create “tiny pores” on the cell membrane. Then, μsLVPEF was applied to “expand and control” the size of pores to promote the effective fusion of cells.

Based on pulse power technology, we developed a cell fusion instrument. Then, several experiments were carried out. ① Microscopic angle: μsHVPEF and ns/μsLVPEF with equal energy were applied in fluorescence experiments of cell fusion. The fluorescence reagents Hoechst 33342 and Propidium Iodide (PI) were used to identify fused and dead cells, respectively. The fusion efficiencies based on different pulses can be counted through fluorescence imaging. ② Macroscopic angle: μsHVPEF and ns/μsLVPEF with equal energy were used in cell culture experiments. After cell electrofusion, the cells were placed into a specific medium (in this medium, only the lymphocytes fused with myeloma cells can proliferate). After ten days of cell culture, the visible colony of hybridomas was counted, and the fusion efficiencies were compared. Finally, the following conclusions can be drawn: when compared with the traditional cell fusion method (μsHVPEF), ns/μsLVPEF with the same energy can effectively improve the cell fusion efficiency (approximately 1.5–3.0 times). Cell fusion induced by nanosecond/low-voltage microsecond pulses is a promising method to develop cell fusion technology. 

## 2. Materials and Methods

### 2.1. Ethics Statement and Cell Culture 

The lymphocytes used in each experiment were extracted from the spleens of mice (specific pathogen-free Kunming mice, male, five months old). The feeding and management of mice strictly followed the Chinese Ethical Guidelines for Laboratory Animal Welfare (GB 14925-2001). Mice were provided with adequate food and water. Mice were euthanized by inhaling an overdose of isoflurane when primary spleen-derived lymphocytes were collected. All animal experiments were approved by the Animal Welfare and Ethics Committee of Chongqing Academy of Animal Sciences.

The SP2/0 myeloma cells were purchased from Shanghai Ya Biotechnology. In order to maintain the cell activity, lymphocytes were used within six hours after isolation from the spleens. SP2/0 myeloma cells were cultivated in a 5% CO_2_ humidified atmosphere at 37 °C using RPMI 1640 medium (12633020, Sigma-Aldrich, Rockville, IN, USA).

### 2.2. Cell Electrofusion Platform

**Figure 1:**
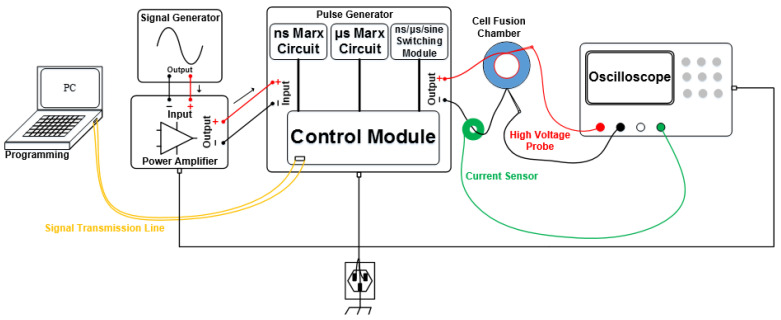
Schematic of cell fusion platform.

**Figure 2:**
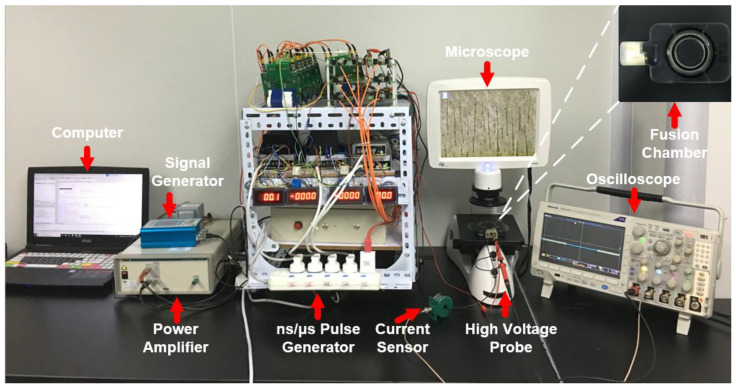
Experimental setups. The voltage is applied to the fusion chamber to make cells fuse.

**Figure 3:**
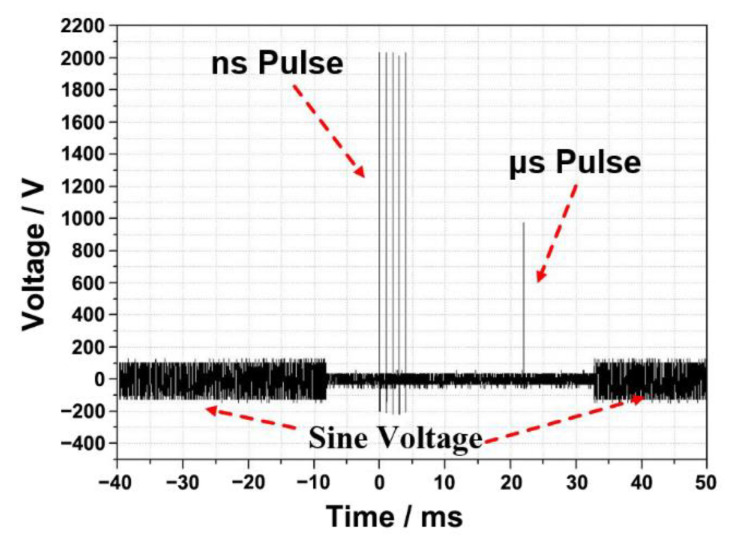
The waveform of the cell fusion instrument. ns/μsLVPEF used in the experiments.

A homemade ns/μsPEF cell electrofusion instrument was designed to set up the cell electrofusion platform. The schematic and experimental setup are shown in Figure 1 and Figure 2, respectively. The signal generator and power amplifier were used to output the sine voltage, causing cells to be arranged in close contact by dielectrophoresis (DEP) [39,40]. There were several modules in the pulse generator to generate different voltage waveforms. Switching between these different modules was carried out with a relay (JPK43A234, WPVAC, Jingdezhen, China), controlled by Field Programmable Gate Arrays (FPGA, Model: ep4ce6, Alinx, Shanghai, China). The output of the cell fusion instrument was connected with a fusion electrode. The current sensor (Model: 2100, Pearson Electronics, Palo Alto, CA, USA) and high-voltage probe (Model: PPE5 kV, Teledyne Lecroy, Chestnut Ridge, NY, USA) were used to collect signals of current and voltage, which were displayed on the screen of the oscilloscope (Model: DPO3024, Tektronix, Shanghai, China). The output parameters of the pulse generator are shown below. Sine voltage—frequency: 0–2 MHz, amplitude: 0–±100 V. Nanosecond pulse—frequency: 0–100 Hz, amplitude: 0–5000 V, pulse width: 0–600 ns, number of pulses: 0–100. Microsecond pulse—frequency: 0–100 Hz, amplitude: 0–2000 V, pulse width: 0–80 μs, number of pulses: 0–100. Figure 3 displays the waveforms of the cell fusion instrument.

### 2.3. Fluorescence Experiments

In order to evaluate the effect of different pulse waveforms from a microscopic angle, fluorescence experiments were developed. In each experiment, 5 × 10^5^ lymphocytes and 5 × 10^5^ SP2/0 cells were used. By utilizing the characteristic of size differences, smaller cells (lymphocytes) rather than larger cells (myeloma cells) were stained with Hoechst 33342 (10 μg/mL, blue fluorescence, Solarbio, Beijing, China) for 15 min at 37 °C to stain the nuclei of lymphocytes. Lymphocytes and myeloma cells were independently washed with phosphate-buffered saline (PBS, Sigma, Beijing, China) twice for 5 min and then washed with fusion medium (osmolarity: 270 to 290 mOsm/L, conductivity: 0.01 S/m, device model: 47-0001, BTX, Holliston, MA, USA) twice for 5 min. After this, two types of cells were transferred into a coaxial fusion electrode (shown in the top right-hand corner of Figure 2, gap: 3.81 mm, device model: 47–0030, BTX, Holliston, MA, USA), waiting for cell electrofusion. Twenty minutes after the electrofusion, PI (1 mg/mL, red fluorescence, Life Technologies, Waltham, MA, USA) was added to the cell suspension and incubated for 6 min. Four visual fields were randomly selected to observe the fluorescence images by fluorescence microscopy (Life Technologies, Waltham, MA, USA), and the multiple channels of fluorescence images were used to calculate the efficiency of cell fusion (cell fusion rate = number of multinucleated cells/total number of myeloma cells). When the blue fluorescence was observed in a large cell, it indicated that the two types of cells had fused. The dead cells (stained with the PI fluorescent molecule) were excluded from cell fusion efficiency analysis. 

### 2.4. Hybridoma Culture Experiments

The cell processing in culture experiments was similar to that previously described, except that no fluorescence dye was added. In order to compare the efficiency of cell fusion more intuitively, we carried out hybridoma culture experiments. After electrofusion, the cells were transferred into a specific medium—Clona Cell™ semisolid culture medium (Model: 03804, Stemcell, Vancouver, BC, Canada)—in which only fused hybrid cells could survive and proliferate, and the other cells would die. Ten days later, the colonies of cells could be counted visibly by the naked eye.

### 2.5. Definition of Cell Fusion Efficiency

In fluorescence and culture experiments, cell fusion efficiency was determined as the ratio between the number of hybrid cells and the number of SP2/0 cells between the electrodes [18].

### 2.6. Electrofusion Protocols

**Figure 4:**
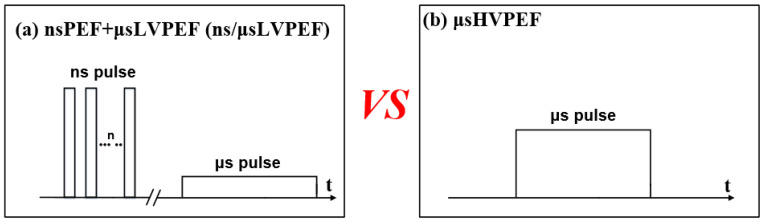
Schematic of pulses used in the experiments. (**a**) The energy dose of ns/μsLVPEF is the same as that of (**b**) μsHVPEF.

ns/μsLVPEF and μsHVPEF were applied to the cell suspension. The energy dose of ns/μsLVPEF was the same as that of μsHVPEF. The electrical dose was used to facilitate comparison, as described by the following equation [41,42]:

(2) $$Dose=\overset{N}{\underset{n=1}{\sum }}E_{n}^{2}\times T_{n}\left[kV^{2}\mu s/cm^{2}\right]$$

(3) $$E_{N}=U_{n}/d$$

where *E_n_* is the electric field intensity of the *n_th_* pulse, *T_n_* is the duration of the *n_th_* pulse measured by an oscilloscope, and *N* is the total number of pulses. *U_n_* is the voltage of the *n_th_* pulse measured by the oscilloscope. *d* is the electrode gap. The schematic of the pulses applied in experiments is shown in Figure 4.

### 2.7. Statistical Analysis

All data are presented as the means ± standard deviation (SD) of more than four independent experiments, and the significance of the indexes between the different parameter groups was tested. OriginPro (Version: 9.0, OriginLab^TM^, Northampton, MA, USA) was used to analyze the statistically significant differences between the sham control and other experimental groups.

## 3. Results

The purpose of this paper is to compare the cell fusion efficiencies of μsHVPEF with those of ns/μsLVPEF. There were three independent experimental groups, and each group contained three pulsing conditions (ns/μsLVPEF, μsHVPEF, control). The field strength of microsecond pulses in each group was different. All experimental results were compared within the groups, not between groups. All pulse parameters used in the experiments are shown in Table 1.

### 3.1. Fluorescence Experiments

**Figure 5:**
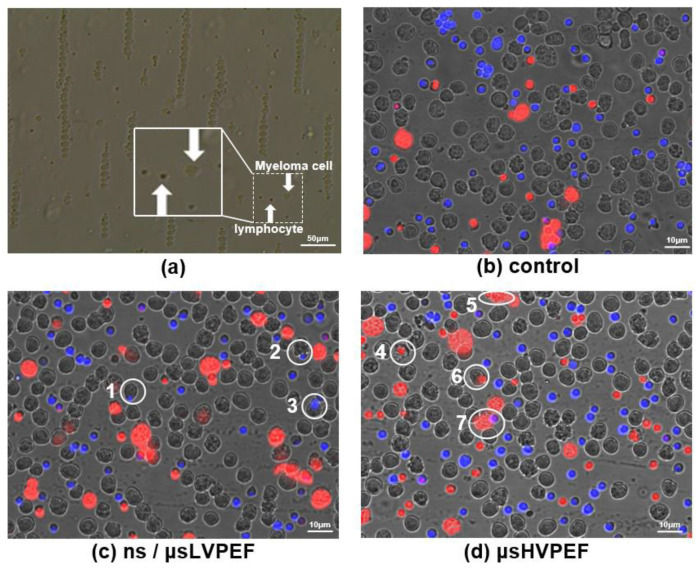
Fluorescence imaging of group 1 based on lymphocytes and myeloma cells. (**a**) Cells are arranged in pearl chains by sine voltage, which will establish close contact between cells. The myeloma cells are much larger than lymphocytes. (**b**) Control group: no pulse voltage is applied. (**c**) Cell fusion by applying ns/μsLVPEF. Blue fluorescence represents nuclei of lymphocytes, and red fluorescence indicates dead cells. (**d**) Cell fusion based on μsHVPEF. The energy dose of μsHVPEF is the same as that of ns/μsLVPEF.

**Figure 6:**
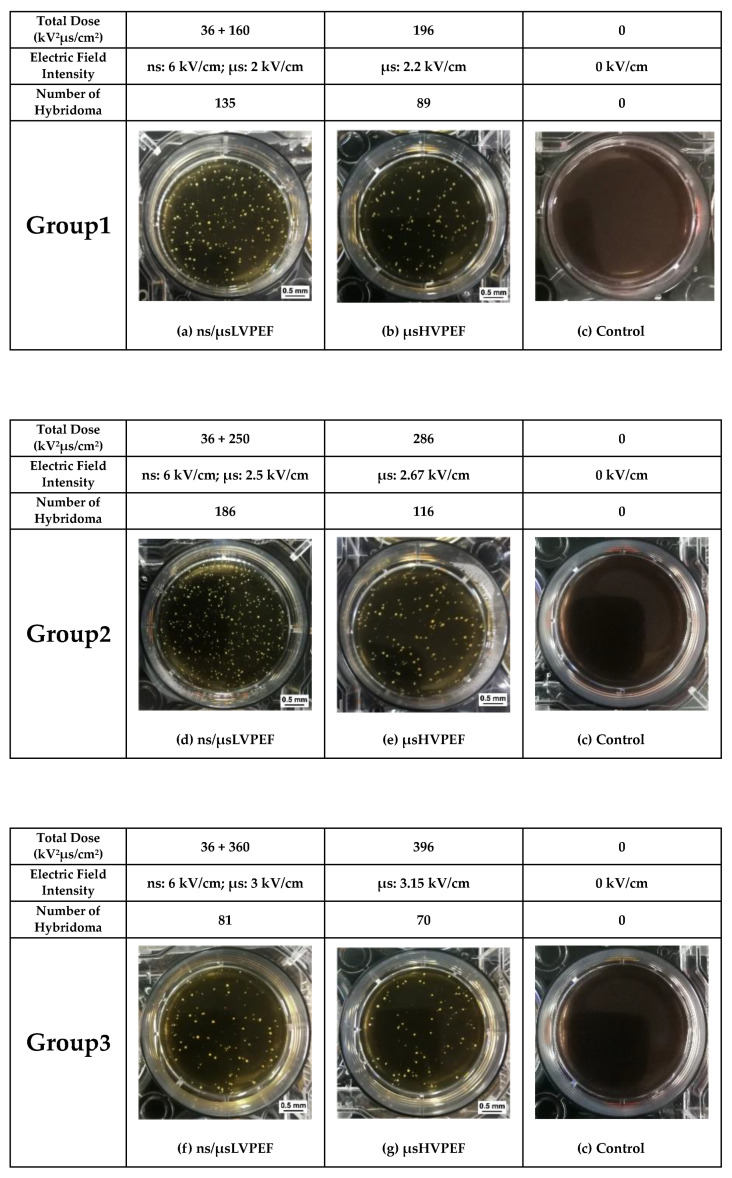
Culture experiments based on lymphocytes and myeloma cells. After cell fusion, the cells were transferred into six-well plates to proliferate for ten days. The white cell colonies in the field of vision represent hybridoma.

**Figure 7:**
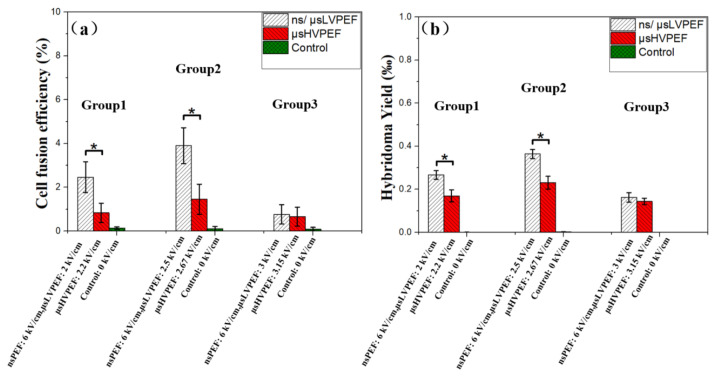
Statistical data of fluorescence experiments and culture experiments (* *p* < 0.05). (**a**) Cell fusion efficiency of fluorescence experiments. Each group contains ns/μsLVPEF, μsHVPEF, control. (**b**) Hybridoma yield of culture experiments. White, red, green represent the data of ns/μsLVPEF, μsHVPEF, control, respectively.

Nuclei of the smaller-sized cells (lymphocytes) were stained with the blue fluorescent molecule to judge whether or not the two cells were fused. Small-sized cells with inconspicuous nuclear membraned under fluorescence microscopy (the small cell is in the larger cell) were judged as fusion cells (Supplementary Figure S1). The merged fluorescence images are displayed in Figure 5, and the white circles labeled with numbers (1–7) represent the fused cells. After fusion, PI was used to distinguish the dead cells (which could not be included in cell fusion efficiency analysis) from the cell suspension. The growth of hybridoma cells is shown in Figure 6. The statistical data of the fluorescence experiments are shown in Figure 7a. In group 1, the cell fusion efficiencies of different parameters were 2.46%, 0.83%, 0.14%, respectively. In group 2, the cell fusion efficiencies were 3.90%, 1.45%, 0.11%, respectively. According to group 1 and group 2, there was a significant difference in fusion efficiency between ns/μsLVPEF and μsHVPEF, and the efficiency of ns/μsLVPEF was approximately 2.5–3.0 times that of the equal energy dose in μsHVPEF. In group 3, the cell fusion efficiencies of different parameters were 0.76%, 0.66%, 0.09%, respectively.

### 3.2. Culture Experiments

After cell electrofusion, the cells were transferred into a six-well plate filled with a culture medium. After ten days of incubation at 37 °C, hybridoma visible to the naked eye could be counted, which are displayed in Figure 6. The hybridoma yields of ns/μsLVPEF, μsHVPEF, and control are shown in Figure 7b. In group 1, the hybridoma yields of different parameters were 0.27% (135 cells), 0.17% (85 cells), and 0%, respectively. In group 2, the hybridoma yields were 0.37% (185 cells), 0.23% (115 cells), and 0%, respectively. In group 1 and group 2, we observed a significant improvement in hybridoma yields after applying ns/μsLVPEF. The efficiency of ns/μsLVPEF was approximately 1.50–1.56 times higher than that of μsHVPEF. In group 3, the hybridoma yields were 0.16% (80 cells), 0.14% (70 cells), and 0‰, respectively. However, there was no significant difference between ns/μsLVPEF and μsHVPEF in group 3. We believe that this may be due to the respective properties of the two electric fields, which we will analyze in the Discussion section.

## 4. Discussion

Fusion efficiency affects the number of hybridomas. Kao et al. found that PEG can be used for the fusion of protoplasts [43] and then widely used in the preparation of hybridomas [44,45,46]. However, hybridoma technology involves the fusion of immune cells and cancer cells. Immune cells can be affected by the toxicity of PEG and die. PEG fusion will result in some potential antibodies not being discovered. Zimmermann et al. created a method to fuse cells with a unipolar pulsed electric field, which provides new ideas for improving the fusion efficiency and enhancing the reproducibility of operations [22].

H. Weber et al. found that the fusion of protoplasts with pulsed electric fields can significantly increase the number of clones, with higher fusion efficiency than PEG [47]. U Karsten et al. demonstrated that the electrofusion method could increase the efficiency of hybridoma production by up to 53 times. In addition, the hybridomas prepared by electrofusion grew more vigorously and could form monoclonal colonies visible to the naked eye earlier [48]. In order to further improve the fusion efficiency of traditional electrofusion methods, we propose, for the first time, the combined application of ns/μsLVPEFs to improve traditional electrofusion methods.

After the comprehensive analysis of the effects of ns/μsLVPEF and μsHVPEF on cell fusion, hybridoma culture and fluorescence experiments showed that ns/μsLVPEF could improve the efficiency of cell fusion. Several questions need to be discussed: What is the difference between ns/μsPEF and other pulses (μsPEF and nsPEF)? What is the improvement mechanism of cell fusion efficiency based on ns/μsLVPEF?

### 4.1. Characteristics of Cell Fusion Based on μsPEF (μsHVPEF or μsLVPEF)

Advantages: The more comprehensive the pulse width, the stronger the ability to expand the pores [42]. The dynamic development of pores is related to the electric field force. Three types of electric force can be induced by external electricity, which can be expressed by the formula below [49]:

(4) $$F=\rho E+\frac{1}{2}E^{2}\nabla \varepsilon +f$$

Among them, *F* is the electric field force, *ρ* is the space charge, *E* is the external field strength, *ε* is the dielectric constant, and *f* is the strain force of electrostriction. According to the law of conservation of momentum, *F* × *t = m* × *v*, where *t* is the pulse width, *m* is the mass of the phospholipid bilayer, and *v* is the displacement velocity of the lipid layer. The displacement of the phospholipid bilayer can be equivalent to the diameter of pores on the cell membrane. Combined with formula (4) and the law of conservation of momentum, the radius of the pore can be calculated by the following formula:

(5) $$r=\frac{\int_{0}^{t}vdt}{2}=\frac{\int_{0}^{t}\frac{Ft}{m}\;dt}{2}=\frac{2\rho Et^{2}+\nabla \varepsilon E^{2}t^{2}+2ft^{2}}{8m}$$

It can be seen from the formula that the pulse width contributes the most to the development of the pore size. For example, the radius of the pore electroporated by nsPEF and μsPEF was calculated, respectively. The parameters of nsPEF were electric field intensity 6000 V/cm and pulse width 200 ns. The parameters of μsPEF were electric field intensity 2000 V/cm and pulse width 40 μs. The radius of the pores generated by nsPEF was (6 × 10^−11^ ×$\;\frac{\rho }{m}$ + 1.8 × 10^−7^ ×$\;\frac{\nabla \varepsilon }{m}$ + 1 × 10^−14^ ×$\;\frac{f}{m}$). The radius of the pores generated by μsPEF was (8 × 10^−7^ × $\frac{\rho }{m}$ + 8 × 10^−4^ × $\frac{\nabla \varepsilon }{m}$ + 1 × 10^−10^ × $\frac{f}{m}$). It can be seen from the calculation that the pores generated by μsPEF were much larger than those by nsPEF. The experiments also proved that the pore size could be increased by choosing a wider pulse width [50].

Disadvantages: Since the transmembrane potential induced by the external microsecond pulses is proportional to the diameter of the cells, it is difficult to fuse the different-sized cells. In Figure 5d, the highlighted cells (white circles 4, 5, 6, and 7) represent fused cells. These cells are stained with PI (dead cells), so they cannot be counted in the fusion efficiency measurement. It is challenging to ensure that two types of cells are simultaneously in the optimal electroporation state. Failure to address this can lead to fusion failure or cell death. Therefore, the cell fusion efficiency is low when applying μsPEF.

### 4.2. Characteristics of Cell Fusion Based on nsPEF

Advantages: In the best case for cell fusion, the cell electroporation should mainly concentrate on the cell contact zone, and the remaining zones of the cell membrane should have no or few pores. This can be achieved by applying nsPEF [34]. Using formula (6) for the time constant, the phenomenon of concentrated electroporation can be explained. *C* is the membrane capacitance. *Se* and *Si* denote the conductivity of the extracellular and intracellular fluid, respectively. The cell contact zone is surrounded by a high-conductivity cytoplasm (0.25 S/m) on both sides [51]. As for the rest of the cell membrane, there is high-conductivity cytoplasm (0.25 S/m) on one side and low-conductivity extracellular cell fluid (0.01 S/m, which is commonly used in cell fusion research [52,53], including our experiments) on the other side. The charging time of the contact zone is much shorter than those of other areas, and it is easier to reach the electroporation threshold of the contact zone. Consequently, the electroporation will be mainly concentrated in the cell contact zone when using nsPEF.

(6) $$\tau =rC\left(\frac{1}{2se}+\frac{1}{si}\right)$$

Disadvantages: The cell fusion process will take several minutes [54]. In this process, the pores are required to remain “open” until the cell fusion is complete. Research [55] showed that nsPEF did not increase the size of pores formed in the membrane. Similarly, Si et al. found that short high-voltage sub-microsecond pulses also contributed less to pore development and only produced tiny pores, which could recover quickly after the pulses [24]. The efficiency of cell fusion will be limited by nsPEF due to the tiny and easily recoverable pores.

### 4.3. Cell Fusion Based on ns/μsLVPEF

**Figure 8:**
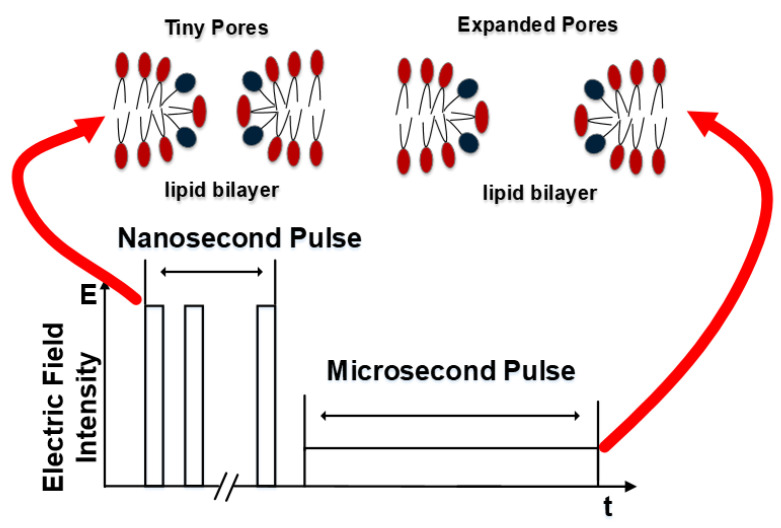
Basic principle of ns/μsLVPEF. Tiny pores are created by nsPEF, and μsLVPEF is used to expand the pores.

The advantages and disadvantages of nsPEF and μsPEF have been analyzed above, and we combined the advantages of these two pulses to offset the shortcomings of each type. The basic principle of ns/μsLVPEF is shown in Figure 8. The nsPEF is equivalent to pretreatment. According to formula (5), as long as there is an energy input, the size of the pores will continue to increase. When applying nsPEF, the tiny pores created in the cell’s contact zone are not large enough for cell fusion [29]. After this, the pores can be expanded by the following μsLVPEF, resulting in cell fusion. According to Figure 7, we can see that there is a significant difference between ns/μsLVPEF and μsHVPEF when the electric field intensity of μsPEF is of a medium or low level (group 1, group 2). However, if the electric field intensity of μsPEF increases excessively (group 3), there is no difference between ns/μsLVPEF and μsHVPEF. This is because some cells are dead in group 3 due to irreversible electroporation.

This study showed that a synergistic effect was evoked by combining nanosecond pulses with microsecond pulses, which improved the cell fusion efficiency. A cell electrofusion platform based on pulse power technology was built to study the effectiveness of this pulsing method. We evaluated the effect of different pulses on cell fusion efficiency from two perspectives: fluorescence experiments (microscopic angle) and culture experiments (macroscopic angle). According to the law of equal energy, the cell fusion efficiencies of ns/μsLVPEF and μsHVPEF were compared. In summary, we can conclude that, compared with μsHVPEF, cell fusion efficiency can be raised 1.5–3.0 times when applying ns/μsLVPEF. The regulation mechanism and distribution of electroporation when applying ns/μsLVPEF are unclear, and further investigations are warranted.

## Figures and Tables

**Table 1:** The pulse parameters used in the experiments.

Group	Pulse Type	ns Electric Field (kV/cm)	ns Pulse Width (µs)	ns Number	μs Electric Field (kV/cm)	μs Pulse Width (µs)	μs Number	Total Dose (kV^2^μs/cm^2^)
1	ns/μsLVPEF	6	0.2	5	2	40	1	36 + 160
	μsHVPEF	0	0	0	2.2	40	1	196
	Control	0	0	0	0	0	0	0
2	ns/μsLVPEF	6	0.2	5	2.5	40	1	36 + 250
	μsHVPEF	0	0	0	2.67	40	1	286
	Control	0	0	0	0	0	0	0
3	ns/μsLVPEF	6	0.2	5	3	40	1	36 + 360
	μsHVPEF	0	0	0	3.15	40	1	396
	Control	0	0	0	0	0	0	0

## Data Availability

The data that support the findings of this study are available upon reasonable request from the corresponding author.

## Supplementary Materials

The following supporting information can be downloaded at: https://www.mdpi.com/article/10.3390/bioengineering9090450/s1, Figure S1: Comparison of cells in different layers vs. cells in the same layer.
