# Supplementary material for: Substantially Improved Electrofusion Efficiency of Hybridoma Cells: Based on the Combination of Nanosecond and Microsecond Pulses
Source: Bioengineering (Basel). 2022 Sep 7;9(9):450. doi: 10.3390/bioengineering9090450 (PMC9495357; doi:10.3390/bioengineering9090450)
Supplement: Supplementary file 1 [file bioengineering-09-00450-s001.zip › bioengineering-1862644 supplementary for conversion.pdf]

Supplementary Figure

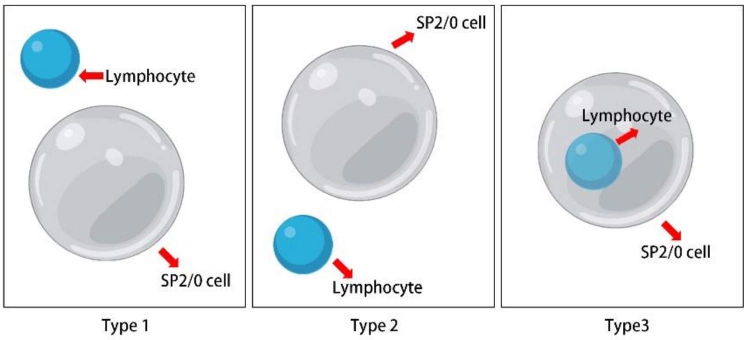

Figure S1. Comparison of cells in different layers vs. cells in the same layer. “Type 1”: The small cell is above the larger one; “Type 2”: The small cell is below the larger one; “Type 3”: The small cell is in the larger one. Only Type 3 is the fused cell.
